# Antibiotic Resistance in Environmental Microbes: Implementing Authentic Research in the Microbiology Classroom

**DOI:** 10.3389/fmicb.2020.578810

**Published:** 2020-10-26

**Authors:** Mangala Tawde, Marianne Williams

**Affiliations:** Queensborough Community College, CUNY, Bayside, NY, United States

**Keywords:** undergraduate research experience, course based undergraduate research experiences, antibiotic resistance, environmental microbiome, community college undergraduate courses

## Abstract

Incorporating Undergraduate Research Experience in Microbiology Classroom. Dr. Mangala Tawde, Associate Professor, Department of Biological Sciences and Geology, Queensborough Community College, CUNY. Undergraduate Research (UR) experience is increasingly being recognized as one of the most transforming experiences students can have in their undergraduate years of education. To make it accessible to all students, incorporating authentic research experiences in the classroom is important and it is a major initiative at Queensborough community college; where we have institutionalized UR as a High Impact Practice. We incorporated an authentic research project into the Microbiology course curriculum for allied health majors. The research project was to isolate and identify antibiotic-resistant microbes from diverse environments. As students are aware of antibiotic resistance being a serious concern in today’s medicine, they get interested and are enthusiastically engaged in the research project. Students collect soil samples from various environments and locations of their choice and then they isolate and identify bacteria that may exhibit antibiotic resistance. The microbes isolated from diverse environments are identified based on the 16s rRNA sequence analysis as well as biochemical tests. The research experience is relevant and aligns well with the course curricula, course learning objectives as well as the college’s General Education objectives.

## Introduction

Inquiry-based team learning is shown to be vital for developing skills such as critical-thinking, scientific problem-solving ability, and acquiring scientific content knowledge in undergraduate biology education (Lord, 2001; Apedoe et al., 2006; Hunter et al., 2007; Kuh, 2008). Many recent studies have shown that research experiences for students early on during their undergraduate years, result in improved learning outcomes, and science career decisions leading to a stronger Science, Technology, Engineering and Mathematics (STEM) workforce (Lopatto, 2004, 2007; Kuh, 2008). Thus Undergraduate Research (UR) experience is considered as one of the best practices to engage and motivate students in undergraduate education (Lopatto, 2004, 2007; Kuh, 2008; Lopatto and Tobias, 2010). Though the traditional one-on-one apprenticeship model with a specific mentor for research internship is known to transform students’ lives and careers, its accessibility is limited to a few students (National Academies of Sciences Engineering and Medicine, 2015). In order to make the pedagogy of undergraduate research accessible to all students, authentic research experiences need to be implemented and incorporated in the undergraduate classroom setting. Thus course-based undergraduate research experiences (CUREs) incorporated in the classroom setting are the response to national “Call for Action” (National Research Council, 2003; American Association for the Advancement of Science, 2011; Ballen et al., 2017) to reform the undergraduate Biology curriculum (Handelsman et al., 2004; Woodin et al., 2010; Lopatto et al., 2011; Wei and Woodin, 2011; Dolan, 2012; Caplan and MacLachlan, 2014; Brownell and Kloser, 2015; Ballen et al., 2017). Students involved in research-based courses are more engaged, more likely to complete their courses, show a greater appreciation of science and inclination toward STEM careers and are more likely to pursue them as compared to those taking traditional courses (Handelsman et al., 2004; Woodin et al., 2010; Lopatto et al., 2011; Wei and Woodin, 2011; Dolan, 2012; Caplan and MacLachlan, 2014; Brownell and Kloser, 2015). There are numerous CUREs that have been proposed as inclusive models to make these experiences accessible to all students (Handelsman et al., 2004; Woodin et al., 2010; Lopatto et al., 2011; Wei and Woodin, 2011; Dolan, 2012; Auchincloss et al., 2014; Caplan and MacLachlan, 2014; Brownell and Kloser, 2015; Brownell et al., 2015; Corwin et al., 2015a,b; Bangera and Brownell, 2017; Mader et al., 2017). However, at institutions without a strong research infrastructure or resources such as community colleges, it is a totally different beast of a challenge for the faculty to convert an entire semester-long course into a CURE. Here we describe a course-based research experience where we incorporated an authentic research experience of studying antibiotic resistance in bacteria isolated from environmental samples into a microbiology lab course that is required for allied health majors.

The student body at Queensborough Community College (QCC) at City University of New York (CUNY) is extremely diverse in its ethnic, cultural and financial backgrounds as well as levels of college preparedness. The unique demographics and needs of CUNY’s community college student population present multiple barriers to students success. Most students come from lower income households, they juggle work, school and family obligations in one of the nation’s most expensive cities. Many have not had science classes in high schools or are returning to school after a hiatus. Understandably, these students are highly unprepared for college-level learning experiences leading to attrition rates of over 30% in our science classes. Therefore incorporating UR experience in classroom is a vital strategy to engage these students, retain and motivate them for rewarding and meaningful educational experiences especially in STEM.

Queensborough CC institutionalized Undergraduate Research (UR) as a High Impact Practice (HIP) in 2013–2014. UR as a HIP is a learning-centered and student centered practice supported by student learning outcomes, assessments, and professional development. Since spring 2014, over 60 faculty members have participated in UR professional development. Close to 100 UR experiences have been offered reaching over 800 students –in addition to the students who engage in the more traditional, dedicated research experiences of the apprenticeship model (QCC Fact book 2018–2019).

The undergraduate research experience in Microbiology course started as a “Research in the Classroom (RIC)” grant initiative that was awarded to M. Tawde by CUNY’s Office of Research. We teach a one-semester Microbiology course (BI 311) that is offered to students seeking to pursue allied health careers and programs. The students typically are rushing to finish the course to get into Nursing, Physician’s Assistant or other programs or may already be in their desired programs. Hence undergraduate research is usually not on their radar and they are not planning to participate in any research program or internship. Most students in our courses have never had any prior UR experience. M. Tawde also teaches one section of Environmental Health class (BI 501) every spring semester. The research experience was implemented in one section of BI 501 and 4 sections of BI 311 lab courses thus involving about 80 students.

### Course Description of Microbiology (BI 311)

A one semester, 4- credit course, Microbiology is intended for Nursing and Allied Health students. The course involves a systematic study of the bacteria, viruses, fungi and helminths with an emphasis on those associated with infectious diseases. Laboratory work includes microbiological techniques and procedures for control.

### Course Description of Environmental Health (BI 501)

A one semester, 4- credit course. An introduction to our environment and its influence on human health; emphasis on scientific principles needed to understand environmental requirements of life; role of air, water, food, energy; studies of effect of human activity on environment and effect of modified environment on human health.

As both classes have a common focus on human health, it is imperative to study the effect environmental microbes may have on human health. Antibiotic resistance is a grave concern in the fields of medicine and healthcare (Allen et al., 2010; Centers for Disease Control and Prevention, 2013; World Health Organization, 2014). Biopharmaceutical agencies are trying to keep up with the growing demand for novel drugs to defeat the antibiotic-resistant pathogens. Hence, we decided to bring this research into our classroom by integrating it into the course curriculum.

Typically in a Microbiology laboratory, students start to learn basic microbiology concepts and standard techniques such as aseptic technique, isolation of bacteria from mixed cultures, staining techniques etc., and then continue to learn how to identify bacteria using Gram staining and various metabolic tests. The midterm practical is conducted over a period of 4–6 weeks and involves identification of “unknown” bacteria. Students learn all the standard “cookbook” microbiology techniques needed to identify the “unknown” bacteria which are actually pure cultures of known bacteria provided to them as unknowns. Thus the students do not receive an authentic research experience.

The research project that we implemented in this course was titled “Research in the Classroom: Antibiotic Resistance in Environmental Microbes.” The goal of the project was to provide an authentic research investigation experience to students as part of their Microbiology laboratory curriculum while they isolate and identify novel microorganisms from the environment and study their resistance/susceptibility to most commonly used antibiotics. Students are aware that antibiotic resistance is a serious concern in the field of health care today. So they are immediately interested and enthusiastic about participating in the research project. The laboratory course syllabi were modified to incorporate the non-traditional activities such as DNA extraction, PCR and DNA analysis by Agarose Gel. A similar CURE has been developed at a larger scale as the PARE project (Genné-Bacon and Bascom-Slack, 2018) as we were developing ours. It is a crowd-sourcing monitoring system that engages students across the country to systematically test and report the prevalence of tetracycline-resistant bacteria from soil at diverse geographic sites. However, our model involved testing antibiotic susceptibility against 12 different antibiotics; not just tetracycline and targets a student population who would not have a research experience otherwise. The majority of students in the above classes usually focus on learning just the techniques but not the concepts behind the techniques or their applications in the real world. Since humans and microorganisms co-exist in dynamic relationships in nature and these relationship critically affects human health; it is crucial that the applications of the microbial genomics are emphasized and understood.

As we implemented the research experience, we attempted to ask more specific questions-

(a) What type of microbes exist at various environments for example soil vs. water vs. surfaces of objects. Do you find more number/types of bacteria in environments with higher human activity as compared to natural environments?
(b) Are the microbes from crowded areas more resistant to antibiotics (or wider variety of antibiotics) compared to those that are isolated from natural environments? Does the environment have any effect on antibiotic susceptibility of organisms that reside in it?

## Materials and Methods

Timeline to incorporate research lab activities during a 15-week semester of a microbiology lab. Laboratory class of Environmental Health class will have similar outline:

**Table:** 

	**Traditional Laboratory Outline**	**Laboratory Outline with implemented research experience**
Week 1	Use and care of the microscope; diversity of microbial life; bacterial shapes	Use and care of the microscope; microbial diversity;Introduction of research project, sample collection.
Week 2	Basic aseptic technique; isolation of single colonies; culturing microbes from the environment; selective and differential media	Basic aseptic technique, culturing environmental samples and isolation of single colonies; selective and differential media
Week 3	Introduction to smear preparation; staining techniques, Gram staining and special stains	Introduction to smear preparation; staining techniques Gram staining and special stains
Week 4	Acid-fast stain; endospore stain**;** Practice for Gram stain	DNA extraction of unknown environment isolates, set up PCR, practice Gram stain
Week 5	Mid-term Lab practical- part 1: Gram stain of unknowns; Inoculate for Metabolic activities	Mid-term Lab practical: Gram stain unknowns and unknown environmental isolates
Week 6	Analysis of metabolic activities, Preparation of dichotomous key for Lab Practical I unknowns	Analysis of metabolic activities, Running Agarose gels, prepare samples for sequencing
Week 7	Physical control of microorganisms: temperature, UV radiation, moisture, Inoculate for Practical I: Part 2 (inoculate metabolic tests)	Physical control of microorganisms: temperature, UV radiation, moisture; Practical I—Part 2 (inoculate metabolic tests)
Week 8	Lab Practical I - Part 2: Analysis of metabolic tests for unknowns, Chemical control of microorganisms: disinfectants and antibiotics	Lab Practical I - Part 2: Analysis of metabolic tests for unknowns, Chemical control of microorganisms: Test for antibiotics resistance
Week 9	Quantification of bacteria in food- milk and chicken broth	Quantification of bacteria in food- milk/chicken broth, Unknowns sequences Analysis
Week 10	Lab reports for unknown due	Lab reports for unknown due

### Description of the Research Activity

The research component was implemented during the spring and fall of 2017 and 2018 semesters. This authentic microbiology wet-lab, hands-on research experience was carried out in groups of 4–5 students each. The students needed to meet twice during the semester outside the class time, (typically during the club hours) each for a block of 1–2 h. These meetings were typically followed by the regular lab class. The first meeting is for DNA extraction and setting up PCR while the second meeting is held to analyze the sequencing data and identification of bacterial species.

Students formulated a hypothesis as to which environmental site may contain the most harmful or highest number of bacteria. Based on their hypothesis, they selected sites for sample collection and went around to swab a small area from the sites such as cafeteria, gym, bathroom, bus-stops, nature trails and botanical garden etc. Some samples came from students’ cell phones. Students were provided with sterile wet swabs to collect the samples of choice. They were asked to bring in the samples at the second class meeting and possibly collect the sample right before the class. After students brought in the soil/surface samples, they streaked them on to sterile Tryptic Soy agar plates and incubated further for growth. At next class meeting, single isolated bacterial colonies were picked and grown in Tryptic Soy broth to confluent cultures. DNA extraction was carried out by using the MoBio DNA PowerSoil DNA isolation kit or Qiagen DNeasy PowerSoil kit and the kit protocols. DNA extraction was followed by setting up a 50 μl polymerase chain reaction (PCR) to amplify the 16s rRNA gene. Once amplified, small amount (10 μl) of amplicons were analyzed by running an agarose gel in the class to ensure amplification of the correct gene product. Remaining amplified product was sent to external sequencing facility GENEWIZ, Inc.,^1^ for sequencing. When the sequencing data was received, it was analyzed using NCBI or DNA Learning Center (DNALC) databases. Students determined the identity of the bacteria by doing a BLAST (Basic Local Alignment Search Tool) search from the National Center for Biotechnology Information (NCBI) database. Alternatively, students used a user friendly version of BLAST – the “DNA Subway” program which is hosted by the DNA Learning Center of Cold Spring Harbor laboratory^2^ (Supplementary Material Part II).

After identifying bacterial species students streaked some of the isolates on the Mueller-Hinton agar plates to form uniform bacterial lawns and carried out Kirby Bauer disk diffusion assay for testing antibiotic susceptibility of select isolates. A BBL disk dispenser was used to dispense commercially available disks impregnated with 12 antibiotics- penicillin, vancomycin, polymyxin B, nitrofurantoin, tobramycin, streptomycin, ciprofloxacin, oxacillin, piperacillin, gentamicin, neomycin, and ampicillin.

All the laboratory procedures were carried out in a BSL 2 laboratory with two hand washing stations, an eye-wash station, an emergency shower, fire blanket etc. Students performed bacterial culturing procedures using aseptic techniques with Bunsen burners and mandatory lab coats. For all bacterial isolates that showed antibiotic resistance, students were supervised closely for all the following procedures performed.

The students submit a comprehensive lab activity report at the end of the semester. The entire research project makes up 10% of the course grade for the students. Other course sections involve a variety of other course activities since 10% of the course grade is at the discretion of the individual instructor.

### Guidelines for Writing the 10% Project Report

• What was the research project that you participated in?
{Antibiotic resistance (susceptibility) of environmental microbes}
• Describe the procedures and methods
• Sample Collection- location (where did you pick your sample from? Home/outside/kitchen/cafeteria/Gym etc.) How did you collect sample? (By swabbing/picking soil?)
• Growing bacteria (You streaked the swab on an TSA agar plate and incubated it for 24–48 h)
• Genomic DNA extraction by using a MoBio PowerSoil/Qiagen DNeasy PowerSoil kit (describe briefly)- 1–2 paragraphs
• Kirby-Baur assay for Antibiotic testing- which antibiotics did you test for? Which antibiotics was your bacterium found to be sensitive or resistant to?
• Analysis/viewing of genomic DNA or PCR amplified 16s rRNA product on Agarose gel by gel Electrophoresis
• What are your thoughts about the research project? (Interesting/Not Interesting/Hmm?)

### Surveying Students’ Attitudes Toward the CURE

Though we were not able to perform a formative assessment of the impact of integrating research experience into the course, students were surveyed for their attitudes toward and feedback about their UR experience using following questions/reflection pointers -

1. The Research project as UR experience helped me understand the course material better.
2. I think I can apply the learned knowledge to newer concepts.
3. After participating in the Research, I am able to comprehend my course material better.
4. How much did the research experience help you to integrate the course concepts in your learning process?
5. How much do you think the course materials were integrated into the research project?
6. How well do you think the course materials were integrated into the research project?
7. How did you like doing the research activities in hands-on form/in laboratory?
8. How did you like doing the research activities online, downloading information from other resources?
9. Has your appreciation for science as it relates to everyday life increased?
10. Would you like to participate in a science research project in other classes at QCC?

## Results

**FIGURE 1:**
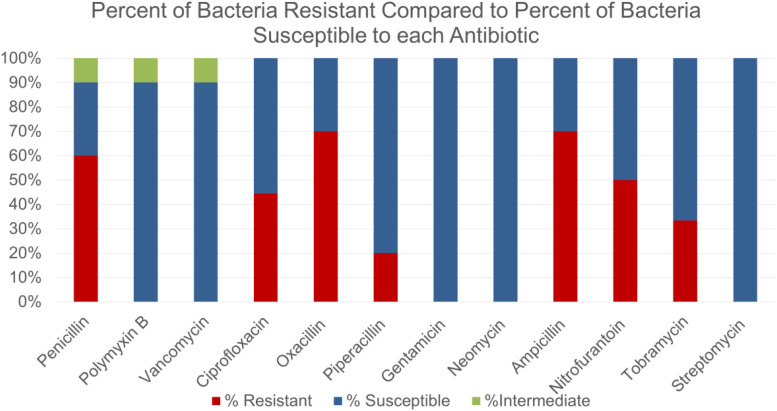
Comparing the susceptibility or resistance of environmental isolates against antibiotics most commonly tested in Microbiology laboratory.

Some of the bacterial species identified were not surprisingly those commonly found on human skin such as *Staphylococcus epidermidis, Staphylococcus aureus* and *Staphylococcus haemolyticus.* Other bacteria that were isolated included various strains of *Bacillus subtilis, Bacillus cereus* and *Escherichia coli.* Some novel species such as *Staphylococcus caprae, Bacillus circulans* were identified as well. Students were intrigued to observe that majority of isolates showed high resistance to many commonly used antibiotics such as penicillin, oxacillin and ampicillin (Figure 1). However, bacteria isolated from crowded places were not necessarily found to be more resistant to tested antibiotics (data not shown).

**FIGURE 2:**
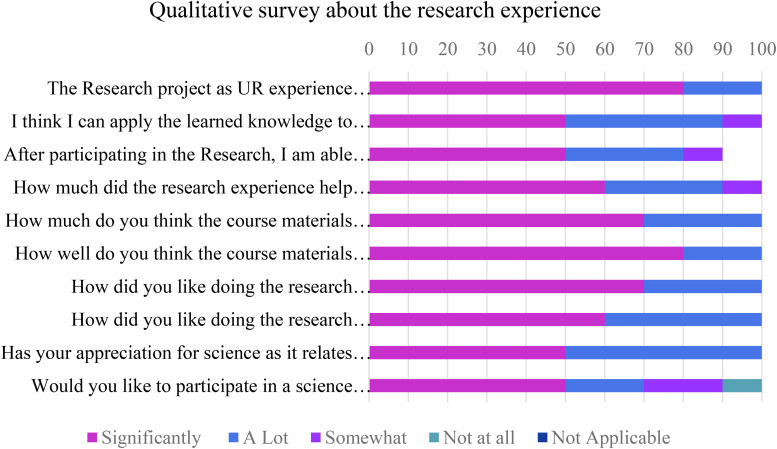
Qualitative student response survey about the research in classroom experience.

Though most students had never had any research experience, all the students in the class displayed mostly positive attitude toward participating in all types of research experiences. Most said they were able to comprehend the course material better, and integrate course concepts in learning process as the concepts were integrated well in the research project. Many liked doing the research activities in hands-on format in laboratory compared to research online or in the library. Their appreciation for science as it relates to everyday life has increased. Most reported that they would like to participate in a science research project in other classes at QCC (Figure 2).

**FIGURE 3:**
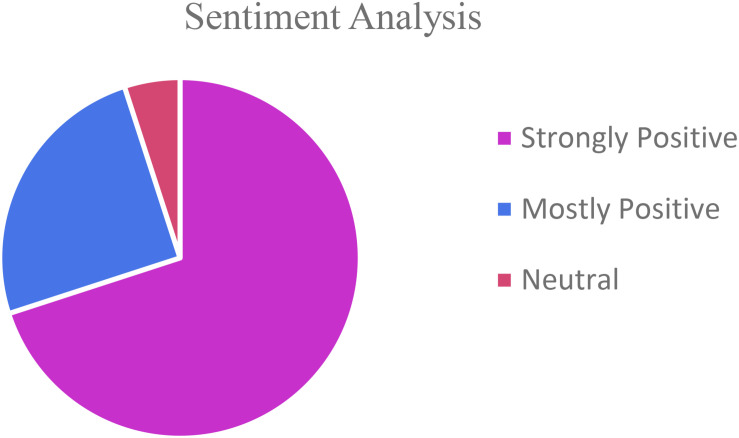
Student Reflections: Sentiment analysis.

Students performed all the laboratory procedures successfully including sample collection, streaking on media plates, isolation and culturing/growing bacteria from the environmental sample, DNA extraction from bacterial isolates, setting up PCR, performing Agarose gel electrophoresis and analyzing the 16s RNA sequence data to identify bacteria isolated from their environmental samples. They displayed increased engagement while learning the procedures and techniques as well as relevance of the research experience to real life situations as is evident from the student response survey (Figure 2) and the student reflections (Figure 3 and Supplementary Material Part II).

Thus the research experience aligned well with the following course learning objectives.

1. Students will understand the general principles of Microbiology with practical emphasis on pathogenic microorganisms.
2. Students will develop the skills necessary to perform various microbiological laboratory procedures.
3. To create an incentive for further investigations in the field and to acquire sufficient background to understand the technical terminology in current publications.
4. To correlate the principles of Microbiology with the students’ own interest and future as a health practitioner.

## Discussion

By integrating a research component directly into an existing Microbiology laboratory course, not just a select few, but ALL students in the class had the opportunity to participate in an inquiry-based real-world application of genomics in Microbiology experience. Incorporating the UR as high impact practice into a course that is required for allied-health career pathway, many students were successfully introduced to biology research concepts and practices, including DNA isolation, amplifying DNA using Polymerase Chain Reactions, DNA sequencing, and genomic/bioinformatics concepts. The vast majority of the students would have never been introduced to these practices had it not been incorporated into a required course. The survey results demonstrate an overwhelmingly positive response and experience for all of the students (Figure 3). The students enjoyed performing the research, recognized the applicability of it to their lives and future careers, and stated that the research experience was valuable. The UR experience helped students make a solid connection between what they learn in class and how it can be applied to the environment around them in real life. It made the students aware of the wide diversity of microbial species in their surroundings as well as introduced them to the technology in the fields of Microbiology and Biotechnology. The students who participated in the project reported significant gain in their knowledge and confidence. They expressed interest in pursuing STEM careers.

Nevertheless, we did face some challenges. There is always time constraint from the instructor point of view as we struggle to “cover” the course content. There is time constraint for students as they are juggling too many classes and work/family responsibilities. These hurdles are prominent especially in community college students. It is extremely challenging to motivate all of the students in a class.

## Conclusion

Here we describe a model CURE that was successfully implemented in a biology lab course at an institution with minimal research infrastructure and limited funding resources. Though it is extremely challenging to incorporate a CURE in a community college science class, it has been a highly rewarding experience for students as we look at the student reflections. It has been a gratifying experience for the faculty as well. We think that this model of CURE can be successfully implemented in other Biology lab courses at other small and large schools alike without too much efforts.

## Data Availability Statement

The raw data supporting the conclusions of this article will be made available by the authors, without undue reservation.

## Ethics Statement

The studies involving human participants were reviewed and approved by City University of New York IRB. The patients/participants provided their written informed consent to participate in this study.

## Author Contributions

MT is the principal and corresponding author on the submission and MW was a contributing author. MT conceived and conceptualized the idea, conducted, organized the research project, carried out the assessment, and wrote the manuscript. MW implemented the research project in her class sections and assisted in writing the results. Both authors contributed to the article and approved the submitted version.

## Conflict of Interest

The authors declare that the research was conducted in the absence of any commercial or financial relationships that could be construed as a potential conflict of interest.

## Footnotes

1 https://www.genewiz.com/

2 https://dnasubway.cyverse.org
