# Supplementary material for: Antibiotic Resistance in Environmental Microbes: Implementing Authentic Research in the Microbiology Classroom
Source: Front Microbiol. 2020 Oct 26;11:578810. doi: 10.3389/fmicb.2020.578810 (PMC7649124; doi:10.3389/fmicb.2020.578810)
Supplement: Supplementary file 1 [file Data_Sheet_1.PDF]

Supplementary Material for--

ANTIBIOTIC RESISTANCE IN ENVIRONMENTAL MICROBES: IMPLEMENTING AUTHENTIC RESEARCH IN THE MICROBIOLOGY CLASSROOM

Mangala Tawde*, Marianne Williams

Queensborough Community College, CUNY

Bayside, NY

Figure 1 Some of the bacterial isolates, were found to be susceptible to most tested antibiotics (A) whereas others were resistant to most of the tested antibiotics (B).

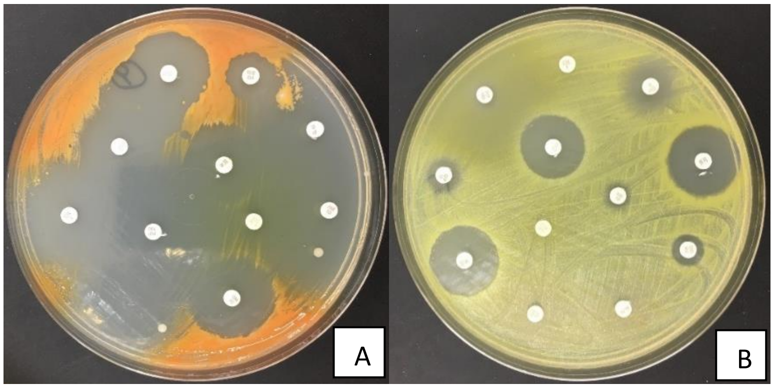

Part I: Examples of Student's quotes from their reflections

a. **Strongly Positive Quote:** I really liked the research activity because it got us curious to find out what is lurking around us and how dangerous it can be. I like hands-on work and it was enjoyable learning about different bacteria. I enjoyed the overall project very much.
b. **Mostly Positive Quote:** I think the research project was very interesting and was relevant to real life. It made us aware of all different types of bacteria that could be present in our surrounding and could make us sick.
c. **Neutral Quote:** I think it was a good project and can be helpful. However it was little hard and took lot of time out of class to complete the project and write the report which was part of the grade.

Part II: DNA Extraction Procedure (From Qiagen DNeasy PowerSoil kit):

A. Obtaining bacteria

1. Using a sterile cotton swab dipped in sterile water swab surface of interest.

2. Streak a Tryptic soy agar plate with the cotton swab cultured from the environment.

3. Incubate at 37°C for at least 24-48 hours or at room temperature for 3-4 days.

B. Bacterial broth

1. Select a colony of interest from the TSA plates streaked with bacteria from the environment.

2. Place colony of interest in Tryptic soy broth.

3. Incubate at room temperature for at least 24 hours.

C. DNA isolation using Qiagen DNeasy UltraClean Microbial Kit

1. Add 1.8ml of microbial culture to a 2ml collection tube and centrifuge at 10,000 x g for 30 seconds at room temperature. Decant the supernatant and spin the tubes again at 10,000 x g for 30 seconds at room temperature. Completely remove the supernatant with a pipette tip.

2. Resuspend the cell pellet in 300ul of PowerBead Solution and gently vortex to mix. Transfer resuspended cells to PowerBead Tube.

3. Add 50ul of Solution SL to the PowerBead Tube.

4. Secure PowerBead Tubes horizontally using the Vortex Adapter tube holder. Vortex at maximum speed for 10 minutes.

5. Make sure the 2 ml PowerBead Tubes rotate freely in the centrifuge without rubbing. Centrifuge the tubes at a maximum of 10,000 x g for 30 seconds at room temperature.

6. Transfer the supernatant to a clean 2 ml collection tube.

7. Add 100ul of Solution IRS to the supernatant and vortex for 5 seconds. Incubate at 4^{0}C for 5 minutes.

8. Centrifuge the tubes at 10,000 x g for 1 minute at room temperature.

9. Avoiding the pellet, transfer the entire volume of supernatant to a clean 2 ml collection tube.

10. Add 900 ul of Solution SB to the supernatant and vortex for 5 seconds.

11. Load about 700 ul into a MB Spin Column and centrifuge at 10,000 x g for 30 seconds at room temperature. Discard the flow-through, add the remaining supernatant to the MB Spin Column, and centrifuge again at 10,000 x g for 30 seconds at room temperature. Each sample processed with require 2-3 loads. Discard all flow-through liquid.

12. Add 300 ul of Solution CB and centrifuge at 10,000 x g for 30 seconds at room temperature.

13. Discard the flow-through. Centrifuge at 10,000 x g for 1 minute at room temperature.

14. Place the MB Spin Column in a new 2ml collection tube.

15. Add 50 ul of Solution EB to the center of the white filter membrane.

16. Centrifuge at 10,000 x g for 30 seconds at room temperature.

17. Discard the MB Spin Column. The DNA is ready for PCR and gel electrophoresis. If PCR is not done immediately the DNA should be stored in a freezer until further use.

D. Polymerase Chain Reaction using Taq PCR Master Mix Kit purchased from Qiagen

Universal 16s rRNA bacterial primers were used as following-

27F (5'-AGAGTTTGATCCTGGCTCAG-3') and 1392R (5'-GGTTACCTTGTTACGACTT-3')

1. Thaw primer solutions and template nucleic acid. Keep on ice after complete thawing and mix thoroughly before use.

2. Thaw Taq PCR Master Mix and mix by vortexing briefly to avoid localized differences in salt concentration.

3. Add 50 ul of Taq PCR Master Mix and 10ul of 10x primer mix to PCR tube.

4. Add 2ul of DNA to the Master Mix and Primer. Vortex briefly to mix the sample.

5. Prepare PCR machine:

Initial step: 94^0C 1minute

35 cycles of the following profile:

Denature step: 94^0C 30 seconds

Anneal step: 54^0C 30 seconds

Extend step: 72^0 45 seconds

Final step to preserve sample: 4^0C ad infinitum

E. Analyze PCR procedures by Gel Electrophoresis using Invitrogen E-gels

1. Load 2-5ul of amplified DNA to e-gel.

2. Run for 8 minutes.

3. Check to see if the DNA was properly amplified.

F. Sequencing done by GENEWIZ

Send amplified DNA to GENEWIZ for sequencing.

G. Analysis of sequence using DNA Subway (dnasubway.cyverse.org)

1. Register at DNA Subway.

2. Use the blue line to determine sequence relationships.

3. Upload the sequence from GENEWIZ for analysis.
